# Risk factors for mortality in hemodialysis patients with COVID-19: a systematic review and meta-analysis

**DOI:** 10.1080/0886022X.2021.1986408

**Published:** 2021-10-11

**Authors:** Fengping Wang, Guangyu Ao, Yushu Wang, Fuqiang Liu, Mulong Bao, Ming Gao, Shulu Zhou, Xin Qi

**Affiliations:** a Department of Nephrology, Chengdu Second People’s Hospital, Chengdu, PR China; b Department of Nephrology, Chengdu First People’s Hospital, Chengdu, PR China; c Department of Cardiology, Chengdu First People’s Hospital, Chengdu, PR China; d Chengdu West China Clinical Research Center Co., Ltd, Chengdu, PR China; e Department of Intensive Care Unit, Chengdu First People’s Hospital, Chengdu, PR China; f Department of Neurology, the Affiliated Hospital of Southwest Jiaotong University & the Third People's Hospital of Chengdu, Chengdu, PR China

**Keywords:** COVID-19, hemodialysis, risk factor, mortality

## Abstract

**Background:**

New evidence from studies on risk factors for mortality in hemodialysis (HD) patients with COVID-19 became available. We aimed to review the clinical risk factors for fatal outcomes in these patients.

**Methods:**

We performed meta-analysis using the PubMed, EMBASE, and Cochrane databases. A fixed- or random-effects model was used for calculating heterogeneity. We used contour-enhanced funnel plot and Egger’s tests to assess potential publication bias.

**Results:**

Twenty-one studies were included. The proportion of males was lower in the survivor group than in the non-survivor group (OR = 0.75, 95% CI [0.61, 0.94]). The proportion of respiratory diseases was significantly lower in the survivor group than in the non-survivor group (OR = 0.42, 95% CI [0.29, 0.60]). The proportion of patients with fever, cough, and dyspnea was significantly lower in the survivor group (fever: OR = 0.53, 95% CI [0.31, 0.92]; cough: OR = 0.50, 95% CI [0.38, 0.65]; dyspnea: OR = 0.25, 95% CI [0.14, 0.47]) than in the non-survivor group. Compared with the non-survivor group, the survivor group had higher albumin and platelet levels and lower leucocyte counts.

**Conclusions:**

Male patients might have a higher risk of developing severe COVID-19. Comorbidities, such as respiratory diseases could also greatly influence the clinical prognosis of COVID-19. Clinical features, such as fever, dyspnea, cough, and abnormal platelet, leucocyte, and albumin levels, could imply eventual death. Our findings will help clinicians identify markers for the detection of high mortality risk in HD patients at an early stage of COVID-19.

## Introduction

Coronavirus disease 2019 (COVID-19), caused by the severe acute respiratory syndrome coronavirus 2 (SARS-CoV-2), has rapidly spread worldwide and has become a global pandemic. As of 19 February 2021, there have been more than 100 million confirmed cases and over 2 million deaths. The common symptoms of COVID-19 include fever, cough, dyspnea, and diarrhea [1]. According to published data, the spectrum of disease is highly variable and can be asymptomatic or progress to fatal multiorgan failure [2]. To date, the mechanisms underlying these differences in disease presentation are not well understood. Multiple international investigators have revealed that patients who are older or have comorbidities, such as diabetes, hypertension, obesity, cardiovascular diseases, and chronic lung disease were not only more susceptible to COVID-19 but also tended to have a higher risk of death due to COVID-19 [3,4]. However, these findings were mainly obtained from studies conducted in the general population. The impact of COVID-19 specifically on hemodialysis (HD) patients is poorly understood.

Patients on maintenance HD with end-stage renal disease (ESRD) are particularly vulnerable to SARS-CoV-2 infection and have a high mortality rate [5]. First, HD patients with significant comorbidities, such as diabetes, hypertension, and cardiovascular disease and older age, place them at higher risk of developing severe illness. Second, HD patients have abnormal immune system responses due to the uremic state [6], which results in both impaired responses and a pro-inflammatory state. Because of their immunocompromised status, the clinical presentation could be different from that of the general population, which may increase the difficulty of diagnosis and treatment of HD patients. Third, due to the nature of their illness, HD patients must travel from home to the hospital routinely and interact with doctors, nurses, medical workers, and other patients in a shared space for at least 12 h weekly, which may lead to widespread cross-contamination.

Previous data revealed that the estimated mortality rate related to maintenance dialysis in patients with COVID-19 ranged between 6.5 and 52% [5,7–11], which is much higher than that in the general population. To effectively predict the progression of the disease and improve protective and preventive strategies, it is crucial to identify the risk factors for mortality in patients with COVID-19 on maintenance HD. Therefore, we aimed to perform a systematic review and meta-analysis of the clinical presentation, disease course, laboratory, outcomes, and risk factors of survivors and non-survivors among HD COVID-19 patients to help clinical physicians make better decisions.

## Materials and methods

### Search strategy

We follow the Preferred Reporting Items for Systematic Reviews and Meta-Analyses statement to perform the meta-analysis [12]. An electronic search of the PubMed, EMBASE, and Cochrane Library databases was conducted from 1 December 2019 to 29 August 2021, with no language restrictions. OAIster and OpenGrey were searched for gray literature. The following keywords and/or medical subject heading terms were used: (‘novel coronavirus’ or ‘2019-nCoV’ or ‘coronavirus disease 2019’ or ‘SARS-CoV-2’ or ‘COVID-19’) AND (HD OR renal insufficiency OR ESRD OR renal replacement therapy OR dialysis OR HD OR chronic kidney disease (CKD) OR chronic kidney failure OR CKD-G5D OR end-stage kidney disease). Details of the search strategy for each database are provided in Supplementary Material 1. A manual search of possible articles relevant to this topic was conducted. We also communicated with the corresponding authors of the included studies for additional data on items needed in our study to accurately calculate the outcome measures.

### Study selection

Two independent investigators (GA and FW) initially screened the titles and abstracts. Full-length articles from the identified studies were retrieved. The inclusion criteria in our meta-analysis were as follows: (1) HD patients with confirmed COVID-19; (2) reported demographics, comorbidities, clinical manifestations, laboratory values, and outcomes of survivors and non-survivors; and (3) risk factors for mortality. Studies were excluded if they were (1) case reports, conference abstracts, editorials, non-clinical studies, and reviews or (2) duplicated publications.

### Data extraction and quality assessment

Two investigators (GA and FW) independently extracted data from the studies that fulfilled our inclusion criteria. Discrepancies were resolved by discussion at group conferences. The extracted data were as follows: name of the first author, study period, study design, region, number of participants, outcomes, HD access, and ESRD vintage. The endpoint was all-cause mortality. The quality of studies was assessed using the Newcastle–Ottawa Scale (NOS) by two independent investigators (YW and QX) [13]. Studies that achieved seven or more, four to six, and fewer than four stars on NOS were considered to be of high, medium, and poor quality, respectively [14]. In addition, we used the Quality In Prognosis Studies (QUIPS) tool for the assessment of the risk of bias [15]. The maximum score was nine stars, and scores greater than six were considered to indicate high quality.

### Statistical analysis

The collected data from the included studies were analyzed using RevMan version 5.3 (The Nordic Cochrane Centre for The Cochrane Collaboration, Copenhagen, Denmark) and Stata software 15.1 (StataCorp LLC, College Station, TX). Reported odds ratios (ORs) and 95% confidence intervals (CIs) were extracted from the included studies. ORs with 95% CIs were used as summary estimates for dichotomous outcomes. In addition, continuous variables were compared by calculating the weighted mean difference (WMD) or standardized mean difference, when applicable. Heterogeneity among studies was evaluated using Cochran’s Q test and *I*^2^ statistic. *I*^2^ statistics were used to assess the magnitude of heterogeneity wherein 25%, 50%, and 75% represented low, moderate, and high degrees of heterogeneity, respectively. The fixed-effect model (Mantel–Haenszel) was used to calculate pooled estimates among studies if *I*^2^ was ≤50%. If *I*^2^ was >50%, the random-effects model (DerSimonian and Laird) was preferred [16,17]. A random-effect model was also applied for the meta-analyses that were analyzed in a fixed-effect model in order to verify our results. Sensitivity or subgroup analyses were conducted to assess the heterogeneity. Sensitivity analysis was performed to investigate the stability of the outcome and was performed by sequentially excluding one study at a time. If there were more than 10 studies, publication bias would be assessed [17]. To visually inspect asymmetry due to publication bias, funnel plots and contour-enhanced funnel plots were constructed. Additionally, Begg’s and Egger’s tests were conducted for the quantitative analysis of publication bias, where *p* < .05 was statistically significant. Statistical significance (*p*) was set at <.05. This study was registered with PROSPERO (number CRD42021241582).

## Results

### Identification of relevant studies

**Figure 1.**
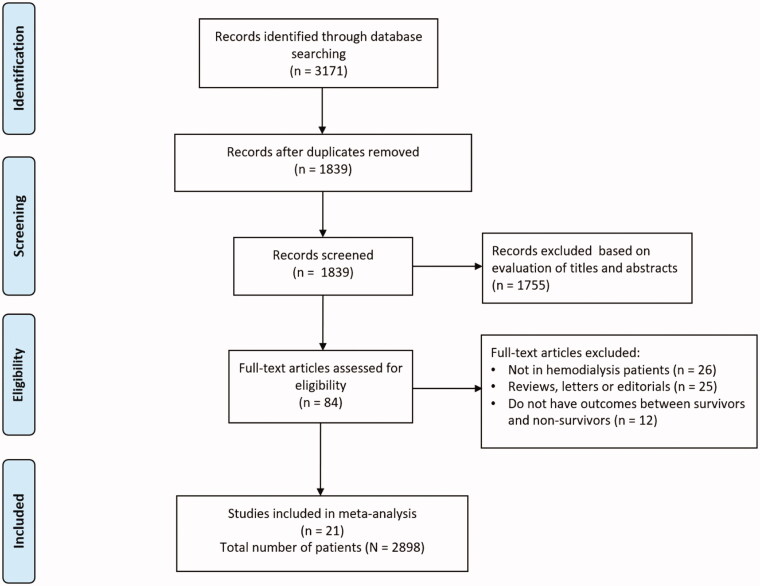
Flow diagram of literature search and study selection.

Through a literature search, a total of 3171 potentially eligible studies were identified based on predefined selection criteria. After removal of duplicates, a review of the titles and abstracts of 1839 articles was performed, and 1755 studies were further excluded after screening the titles and abstracts. A total of 84 articles were obtained and read in full. Of these, 63 studies were excluded for reasons detailed in Figure 1. Ultimately, 21 studies [18–38], comprising 2898 HD patients with COVID-19, were included in this meta-analysis. The process of study retrieval is summarized in Figure 1.

### Study characteristics and quality assessment

Demographic data of the patients in the included trials are presented in Table 1. Among the 21 included studies, two studies were prospective in design, while the others were retrospective. Studies sample sizes ranged from 16 to 741 HD patients with COVID-19. The HD vintage of the patients with ESRD was variable, and the type of angioaccess mostly included arteriovenous fistula and central venous catheter. Table 2 shows the characteristics of the survivor and non-survivor groups, including pre-specified risk factors. The clinical outcome was all-cause mortality, and the overall mortality rate was 19.12%. The details of quality assessment using the NOS tool are presented in Table 3. The quality of the included studies was high, with scores ranging from 7 to 8; the average NOS score was 7.6. According to the QUIPS, for the estimation of quality in the included studies, the evaluation results of each item with potential bias are shown as ‘yes’, ‘partly’, ‘no’, or ‘unsure’ in Table 4.

**Table 1.** Baseline characteristics of included studies.

Author	Country	Research type	Period	Number of patients	ESRD vintage, years^a^		Hemodialysis access			
							Survival		Death	
					Survival	Death	Arteriovenous fistula	Central venous catheter	Arteriovenous fistula	Central venous catheter
Stefan et al. [18]	Romania	Observational retrospective cohort	24 March–22 May 2020	37	2.9 (0.4–5.8)	3.6 (1.8–4.8)	18 (60)	12 (40)	2 (29)	5 (71)
Creput et al. [19]	France	Observational retrospective cohort	13 March–15 April 2020	38	3.2 (0.1–14.2)	4.3 (0.5–17.3)	NR	NR	NR	NR
Zou et al. [20]	China	Observational retrospective cohort	1 January–25 March 2020	66	5.0 (3.2, 6.0)	4.5 (2.2, 7.0)	44 (91.6)	4 (8.4)	16 (88.9)	2 (11.1)
Goicoechea et al. [21]	Spain	Observational retrospective cohort	12 March–10 April 2020	36	NR	NR	NR	NR	NR	NR
Deshpande et al. [22]	India	Observational retrospective cohort	1 March–25 May 2020	75	NR	NR	NR	NR	NR	NR
Bahat et al. [23]	Turkey	Observational retrospective cohort	11 March–12 May 2020	25	NR	NR	NR	NR	NR	NR
Mazzoleni et al. [24]	Belgium	Retrospective cross-sectional cohort	6 March–4 April 2020	40	NR	NR	NR	NR	NR	NR
Seidel et al. [25]	Germany	Observational retrospective cohort	February–April 2020	56	NR	NR	NR	NR	NR	NR
Min et al. [26]	China	Observational retrospective cohort	Until 28 February 2020	74	5.6 (3–7.1)	4.3 (2.4–4.9)	43 (71.0)	17 (29.0)	9 (61.5)	5 (38.5)
Sİpahİ et al. [27]	Turkey	Observational retrospective cohort	3 March–23 April 2020	23	NR	NR	NR	NR	NR	NR
Shang et al. [28]	China	Observational retrospective cohort	3 February–4 April 2020	47	NR	NR	NR	NR	NR	NR
Hendra et al. [29]	UK	Observational retrospective cohort	15 April–26 May 2020	148	NR	NR	NR	NR	NR	NR
Sosa et al. [30]	Guatemala	Observational retrospective cohort	1 May–31 July 2020	319	NR	NR	NR	NR	NR	NR
Islam et al. [31]	Turkey	Observational retrospective cohort	NR	34	4.7 ± 3.6	9 ± 7.5	NR	NR	NR	NR
Lugon et al. [32]	Brazil	Observational retrospective cohort	February–December 2020	741	NR	NR	469 (77.9)	133 (22.1)	86 (61.9)	53 (38.1)
Turgutalp et al. [33]	Turkey	Observational retrospective cohort	17 April–1 June 2020	567	NR	NR	NR	NR	NR	NR
Ahmed et al. [34]	United Arab Emirates	Observational retrospective cohort	1 March–1 July 2020	152	NR	NR	NR	NR	NR	NR
Can et al. [35]	Turkey	Observational retrospective cohort	1 January–30 December 2020	35	NR	NR	NR	NR	NR	NR
Medjeral-Thomas et al. [36]	UK	Observational retrospective cohort	March–May 2020	106	NR	NR	NR	NR	NR	NR
Prasad et al. [37]	India	Observational prospective cohort	15 March–31 July 2020	263	NR	NR	162 (71.1)	66 (28.9)	16 (45.7)	19 (54.3)
Quiroga et al. [38]	Spain	Observational prospective cohort	15 March–28 April 2020	16	NR	NR	6 (50)	6 (50)	2 (50)	2 (50)

^a^ Data presented as median (IQR) or mean (SD); NR: not reported

**Table 2.** Patient characteristics of included studies.

Author	Age^a^		Male (%)		Diabetes		Hypertension		Cancer		Cardiovascular disease				Respiratory disease			
											Coronary heart disease		Ischemic cardiopathy		COPD		Chronic lung disease	
	Survival	Death	Survival	Death	Survival	Death	Survival	Death	Survival	Death	Survival	Death	Survival	Death	Survival	Death	Survival	Death
Stefan et al.	63 (55–68)	69 (55–72)	16 (53)	3 (43)	11 (37)	2 (29)	25 (83)	5 (71)	1 (3)	1 (14)	13 (43)	6 (86)	NR	NR	1 (3)	2 (29)	NR	NR
Creput et al.	65 (31–89)	74 (63–85)	22 (73)	8 (100)	15 (50)	2 (25)	29 (97)	7 (88)	NR	NR	NR	NR	12 (40)	5 (63)	NR	NR	NR	NR
Zou et al.	65.5 (57.0, 70.5)	60 (52.0, 73.0)	20 (41.7)	11 (61.1)	NR	NR	NR	NR	2 (4.2)	2 (11.1)	10 (20.8)	10 (55.6)	NR	NR	7 (14.6)	3 (16.7)	NR	NR
Goicoechea et al.	69 ± 14	75 ± 6	17 (68)	6 (54)	17 (68)	6 (54)	25 (100)	10 (91)	NR	NR	7 (28)	1 (9)	NR	NR	6 (24)	1 (9)	NR	NR
Deshpande et al.	53.35 ± 12.56	60 ± 11.8	37 (56.1)	6 (66.7)	32 (48.5)	7 (77.8)	49 (74.2)	6 (66.7)	NR	NR	18 (27.3)	4 (44.4)	NR	NR	1 (1.5)	3 (33.3)	NR	NR
Bahat et al.	60.8 ± 14.5	59.4 ± 21.1	9 (36)	1 (20)	15 (75)	3 (60)	15 (75)	4 (80)	NR	NR	7 (35)	2 (40)	NR	NR	1 (5)	0 (0)	NR	NR
Mazzoleni et al.	71 (63–79)	78 (73–82)	14 (48.3)	9 (81.8)	19 (65.5)	7 (63.6)	26 (89.3)	11 (100)	2 (6.9)	1 (9.1)	NR	NR	NR	NR	NR	NR	9 (31.0)	7 (63.6)
Seidel et al.	NR	NR	NR	NR	18 (43.9)	7 (46.7)	34 (82.9)	9 (60.0)	NR	NR	16 (39.0)	5 (33.3)	NR	NR	NR	NR	NR	NR
Min et al.	63.00 (57.00–72.00)	63.00 (59.50–72.00)	25 (41.9)	9 (61.5)	NR	NR	NR	NR	NR	NR	NR	NR	NR	NR	NR	NR	NR	NR
Sİpahİ et al.	NR	NR	NR	NR	8(40)	3 (100)	NR	NR	NR	NR	NR	NR	NR	NR	NR	NR	NR	NR
Shang et al.	57.2 ± 15.0	70.6 ± 11.8	23 (60.5%)	7 (77.8%)	NR	NR	NR	NR	NR	NR	NR	NR	NR	NR	NR	NR	NR	NR
Hendra et al.	61.70 ± 14.6	71.69 ± 11.9	60 (53.6)	24 (66.7)	58 (51.8)	20 (55.6)	91 (81.3)	31 (86.1)	NR	NR	NR	NR	25 (22.3)	18 (50)	NR	NR	11 (9.8)	8 (22.2)
Sosa et al.	NR	NR	NR	NR	68 (29.7)	58 (64.4)	NR	NR	NR	NR	NR	NR	NR	NR	NR	NR	NR	NR
Islam et al.	59.8 ± 13.2	72.8 ± 6.6	12 (42.9)	3 (50)	NR	NR	NR	NR	NR	NR	NR	NR	NR	NR	NR	NR	NR	NR
Lugon et al.	55 ± 16	64 ± 15	364 (60.9)	88 (63.3)	216 (35.9)	77 (55.4)	498 (82.7)	121 (87.1)	21 (3.5)	6 (4.3)	NR	NR	31 (5.1)	10 (7.2)	17 (2.8）	10 (7.2)	NR	NR
Turgutalp et al.	63 (52–71)	66 (57–74)	242 (51.1)	54 (58.1)	218 (46.4)	43 (47.3)	374 (79.1)	70 (79.5)	24 (5.3)	6 (6.5)	NR	NR	180 (42.0)	42 (49.4)	56 (12.7)	21 (23.6)	NR	NR
Ahmed et al.	51.2 ± 11.3	64.1 ± 3.5	112 (81)	11 (79)	75 (54)	3 (21)	NR	NR	NR	NR	NR	NR	NR	NR	NR	NR	NR	NR
Can et al.	NR	NR	9 (37.50)	6 (54.54)	11 (45.83)	8 (72.72)	NR	NR	NR	NR	11 (45.83)	5 (45.45)	NR	NR	NR	NR	NR	NR
Medjeral-Thomas et al.	65 (53–72)	76 (61–80)	59 (66)	7 (44)	48 (53)	9 (56)	NR	NR	NR	NR	NR	NR	NR	NR	NR	NR	NR	NR
Prasad et al.	50.95 ± 13.45	57.00 ± 13.84	146 (64.0)	27 (77.1)	NR	NR	NR	NR	NR	NR	NR	NR	NR	NR	NR	NR	NR	NR
Quiroga et al.	69 ± 17	79 ± 4	9 (75)	4 (100)	4 (33)	3 (75)	11 (92)	2 (50)	NR	NR	2 (17)	0	NR	NR	1 (8)	2 (50)	NR	NR

^a^Age data presented as median (IQR) or mean (SD); COPD: chronic obstructive pulmonary disease; NR: not reported

**Table 3.** Study quality assessment using the Newcastle–Ottawa Scale.

	Selection					Outcome			
Study	Representativeness of exposed cohort	Selection of non-exposed cohort	Ascertainment of exposure	Outcome of interest absent at start of study	Comparability	Assessment of outcome	Follow-up long enough for outcomes to occur	Adequacy of follow-up	Total score
Stefan et al.	*	*	*	*	* *	*	…	…	7
Creput et al.	*	*	*	*	* *	*	…	…	7
Zou et al.	*	*	*	*	* *	*	…	*	8
Goicoechea et al.	*	*	*	*	* *	*	…	…	7
Deshpande et al.	*	*	*	*	* *	*	…	*	8
Bahat et al.	*	*	*	*	* *	*	…	…	7
Mazzoleni et al.	*	*	*	*	* *	*	…	*	8
Seidel et al.	*	*	*	*	* *	*	…	…	7
Min et al.	*	*	*	*	* *	*	…	*	8
Sİpahİ et al.	*	*	*	*	* *	*	…	*	8
Shang et al.	*	*	*	*	* *	*	…	*	8
Hendra et al.	*	*	*	*	* *	*	…	*	8
Sosa et al.	*	*	*	*	* *	*	…	*	8
Islam et al.	*	*	*	*	* *	*	…	*	8
Lugon et al.	*	*	*	*	* *	*	…	*	8
Turgutalp et al.	*	*	*	*	* *	*	…	…	7
Ahmed et al.	*	*	*	*	* *	*	…	…	7
Can et al.	*	*	*	*	* *	*	…	…	7
Medjeral-Thomas et al.	*	*	*	*	* *	*	…	*	8
Prasad et al.	*	*	*	*	* *	*	…	…	7
Quiroga et al.	*	*	*	*	* *	*	…	*	8

**Table 4.** Quality assessment of included studies based on the Quality In Prognosis Studies (QUIPS).

Quality evaluation of prognosis study						
Study	Study participation	Study attrition	Prognostic factor measurement	Outcome measurement	Study confounding	Statistical analysis and reporting
Stefan et al.	Yes	Yes	Yes	Yes	Partly	Yes
Creput et al.	Yes	Yes	Yes	Yes	Partly	
Zou et al.	Yes	Yes	Yes	Yes	Partly	
Goicoechea et al.	Yes	Yes	Yes	Yes	Partly	
Deshpande et al.	Yes	Yes	Yes	Partly	Partly	
Bahat et al.	Yes	Yes	Yes	Yes	Partly	
Mazzoleni et al.	Yes	Yes	Yes	Yes	Partly	
Seidel et al.	Yes	Yes	Yes	Partly	Partly	
Min et al.	Yes	Yes	Yes	Partly	Partly	
Sİpahİ et al.	Yes	Yes	Partly	Partly	Partly	
Shang et al.	Yes	Yes	Partly	Partly	Partly	
Hendra et al.	Yes	Yes	Yes	Yes	Partly	
Sosa et al.	Yes	Yes	Yes	Partly	Partly	
Islam et al.	Yes	Yes	Partly	Partly	Partly	
Lugon et al.	Yes	Yes	Yes	Partly	Partly	
Turgutalp et al.	Yes	Yes	Yes	Yes	Partly	
Ahmed et al.	Yes	Yes	Yes	Partly	Partly	
Can et al.	Yes	Yes	Partly	Partly	Partly	
Medjeral-Thomas et al.	Yes	Yes	Yes	Yes	Partly	
Prasad et al.	Yes	Yes	Partly	Partly	Partly	
Quiroga et al.	Yes	Yes	Yes	Yes	Partly	

### Demographical characteristics

**Figure 2.**
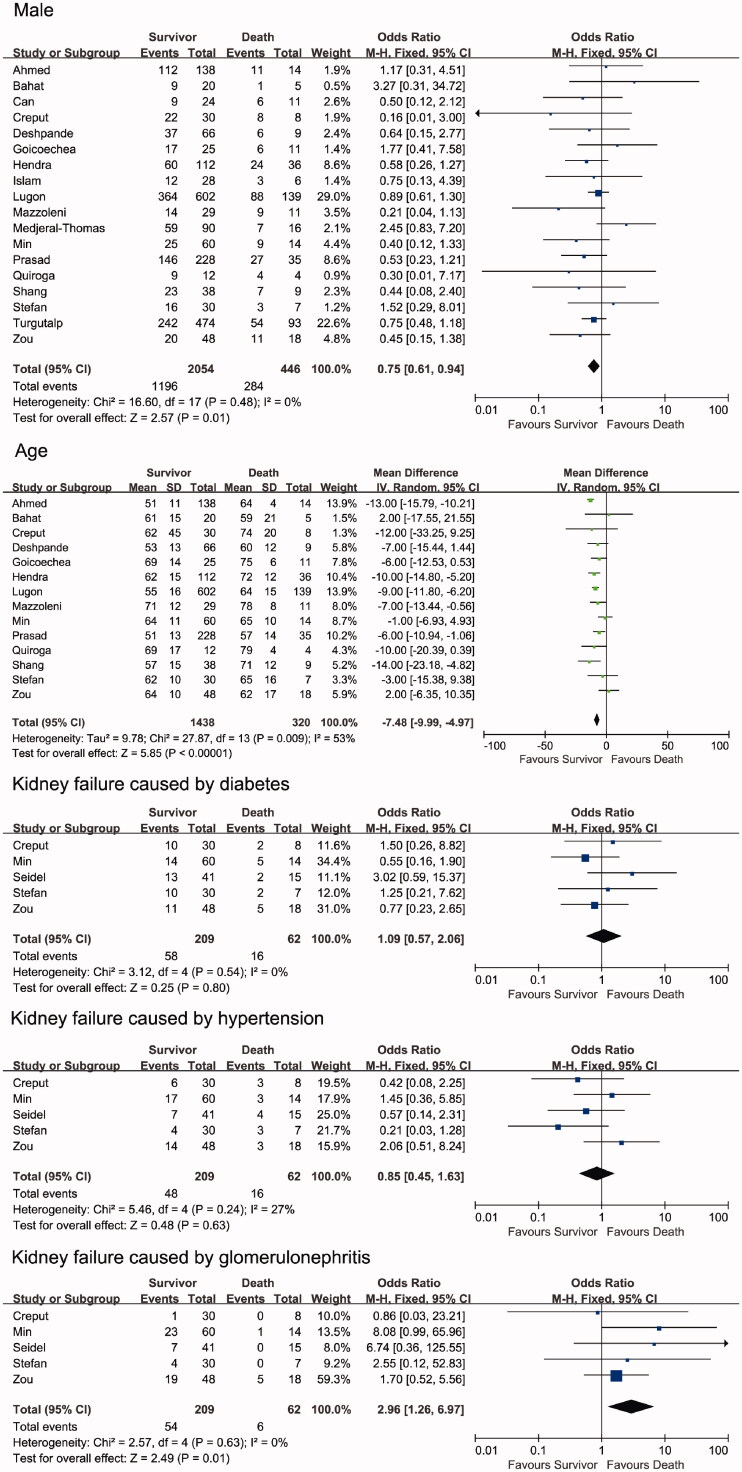
Forest plots depict the comparison of demographical characteristics in survivor and non-survivor groups.

The demographic characteristics of the included studies are shown in Figure 2. The results from the 18 included studies (with a total of 2500 patients) showed that the proportion of males was significantly lower in the survivor group than in the non-survivor group (OR = 0.75, 95% CI [0.61, 0.94], *p* = .01, *I*^2^ = 0%). A random-effects model yielded similar results (Supplemental Figure 1).

The mean age of the patients was 51–71 years in the survivor group across the enrolled studies and 57–79 years in the non-survivor group. Meta-analysis showed that the survivor group was significantly younger than the non-survivor group (WMD = −7.48, 95% CI [–9.99, −4.97], *p* < .00001, *I*^2^ = 53%).

Five studies showed that kidney failure caused by diabetes or hypertension had no significant difference between the mortality and survivor groups (diabetes: OR = 1.09, 95% CI [0.57, 2.06], *p* = .80, *I*^2^ = 0%; hypertension: OR = 0.85, 95% CI [0.45, 1.63], *p* = .63, *I*^2^ = 27%). However, these five studies indicated that the incidence of kidney failure caused by glomerulonephritis was significantly higher in the survivor group than in the non-survivor group (OR = 2.96, 95% CI [1.26, 6.97], *p* = .01, *I*^2^ = 0%). The random-effects model did not alter the overall estimates and yielded results similar to those of the fixed-effect model (Supplemental Figure 1).

### Comorbidities

**Figure 3.**
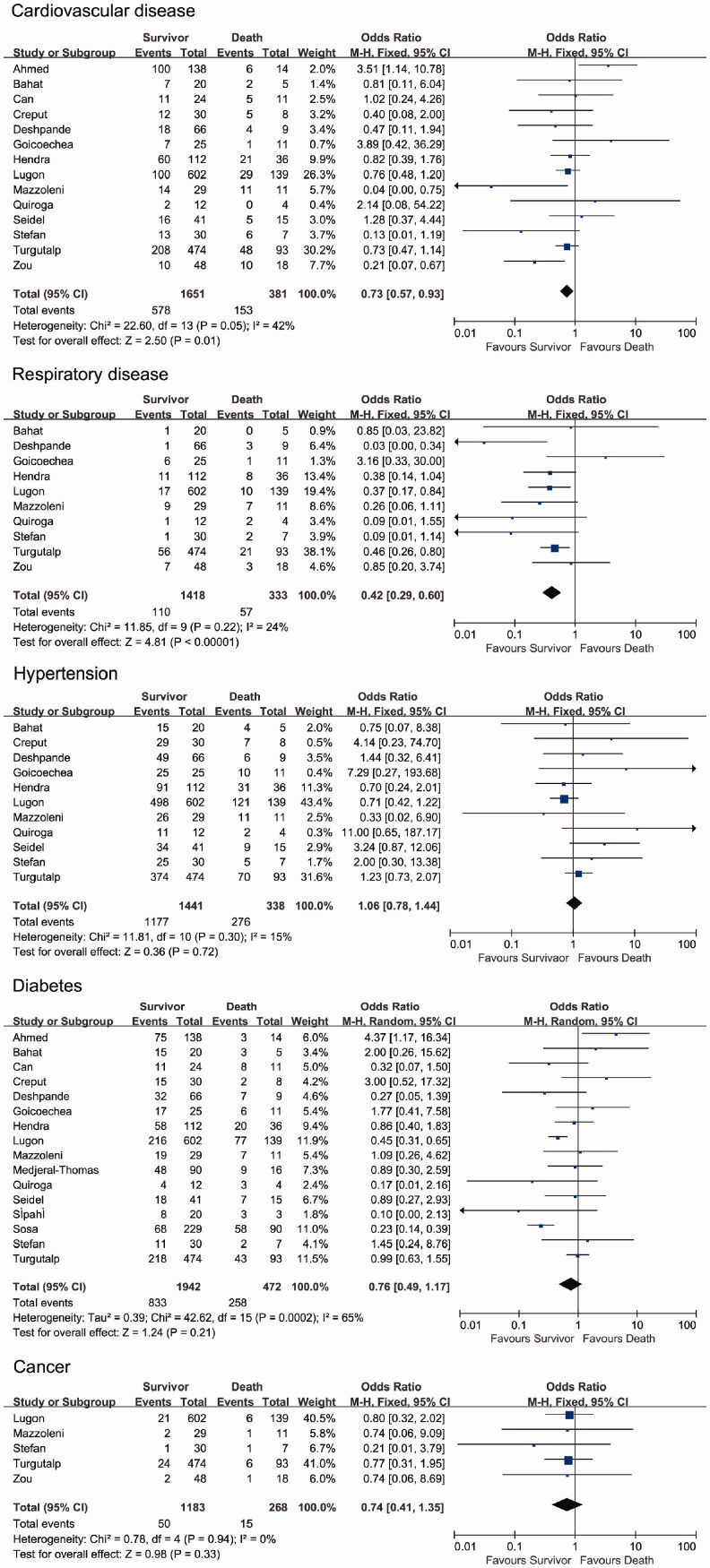
Forest plots depict the comparison of comorbidities in survivor and non-survivor groups.

The comorbidities of the patients in the included studies are shown in Figure 3. The difference in the prevalence of comorbidities was compared between the survivor and non-survivor groups. The proportion of cardiovascular and respiratory diseases was significantly lower in the survivor group than in the non-survivor group (cardiovascular disease: OR = 0.73, 95% CI [0.57, 0.93], *p* = .01, *I*^2^ = 42%; respiratory disease: OR = 0.42, 95% CI [0.29, 0.60], *p*＜.00001, *I*^2^ = 24%). The random-effects model yielded non-significant results for cardiovascular disease but similar results for respiratory disease (Supplemental Figure 1). In addition, meta-analysis showed that the proportion of hypertension, diabetes, and cancer was not significantly different between the survivor and non-survivor groups (hypertension: OR = 1.06, 95% CI [0.78, 1.44], *p* = .72, *I*^2^ = 15%; diabetes: OR = 0.76, 95% CI [0.49, 1.17], *p* = .21, *I*^2^ = 65%; cancer: OR = 0.74, 95% CI [0.41, 1.35], *p* = .33, *I*^2^ = 0%). The random-effects model yielded similar results (Supplemental Figure 1).

### Clinical manifestations

**Figure 4.**
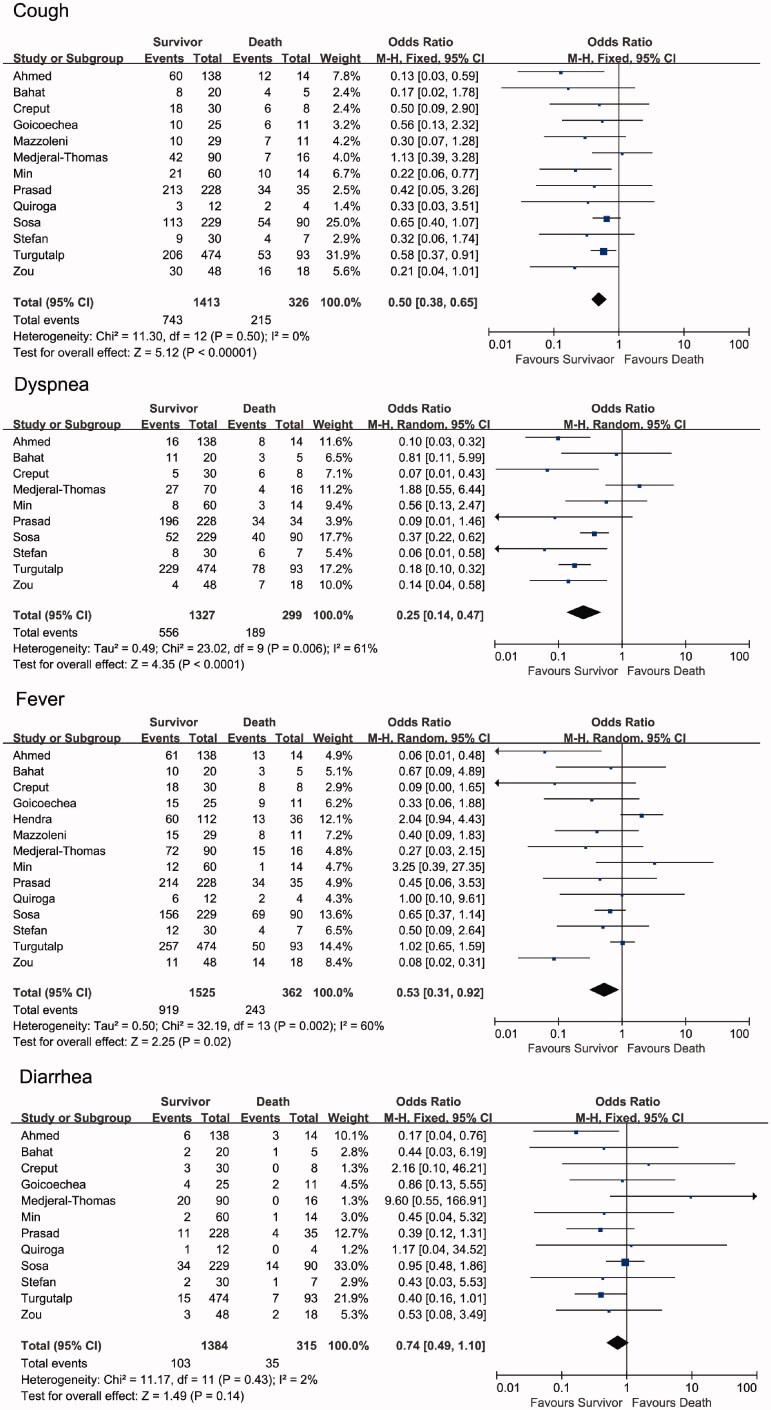
Forest plots depict the comparison of clinical manifestations in survivor and non-survivor groups.

The results of the meta-analysis are presented in Figure 4. Regarding fever, cough, and dyspnea, the proportions were significantly lower in the survivor group (fever: OR = 0.53, 95% CI [0.31, 0.92], *p* = .02, *I*^2^ = 60%; cough: OR = 0.50, 95% CI [0.38, 0.65], *p* < .0001, *I*^2^ = 0%; dyspnea: OR = 0.25, 95% CI [0.14, 0.47], *p* < .0001, *I*^2^ = 61%) than in the non-survivor group. Regarding diarrhea, the proportions were not significantly different between the non-survivor and survivor groups (diarrhea: OR = 0.74, 95% CI [0.49, 1.10], *p* = .14, *I*^2^ = 2%). The random-effects model yielded significant results for both cough and diarrhea (Supplemental Figure 1).

### Laboratory examination

**Figure 5.**
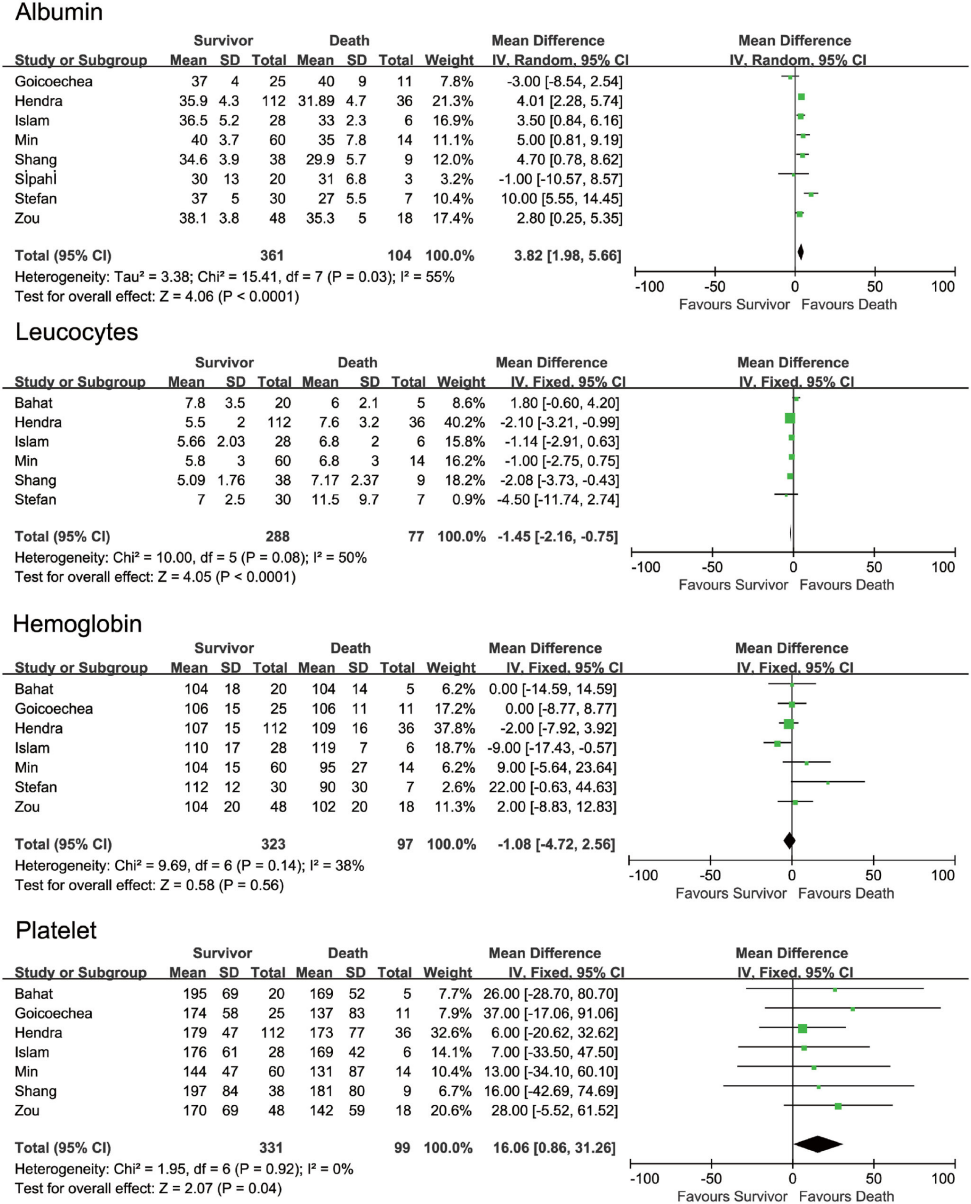
Forest plots depict the comparison of laboratory examination in survivor and non-survivor groups.

As shown in Figure 5, compared with the non-survivor group, the survivor group had higher albumin levels (WMD = 3.82, 95% CI [1.98, 5.66], *p* < .0001, I^2^ = 55%), lower leucocyte counts (WMD = −1.45, 95% CI [–2.16, −0.75], *p* < .0001, *I*^2^ = 50%) and higher platelet counts (WMD = 16.06, 95% CI [0.86, 31.26], *p* = .04, *I*^2^ = 0%). Hemoglobin level and platelet count showed no significant difference between the survivor and non-survivor groups (hemoglobin: WMD = −0.18, 95% CI [−4.72, 2.56], *p* = .56, *I*^2^ = 38%). The random-effects model yielded similar results (Supplemental Figure 1).

### Sensitivity analysis/subgroup analysis and publication bias

Sensitivity analysis was done by excluding one study at a time; subgroup analysis based on countries (European *versus* Asian countries) and sample size (>100 *versus* < 100 patients) did not significantly alter the overall estimates nor reduce the heterogeneity. A funnel plot and contour-enhanced funnel plot representing risk factors, such as sex, age, fever, cough, diarrhea, cardiovascular diseases, diabetes, and hypertension, were compared between the survivor and non-survivor groups. The results were used to evaluate publication bias in this meta-analysis. Based on visual inspection of the funnel plot and contour-enhanced funnel plots alone, there asymmetry was not evident in the analysis of cough as a risk factor, representing a possibility of publication bias. This is further supported by the results of the Begg’s test (*p* = .246), although, the results of the Egger’s test are statistically significant (*p* = .025) (Supplemental Material 2). No publication bias was found in other groups.

## Discussion

Since the mortality rate in HD patients with COVID-19 was much higher than that in the general population [39–41], the aim of this study was to identify the risk factors for mortality associated with COVID-19 in this population. The results of this meta-analysis showed that males and those of older age might have a higher risk of mortality, and comorbidities, such as cardiovascular and respiratory diseases could also worsen the prognosis of COVID-19 in HD patients. Clinical features, such as fever, dyspnea, and cough, may imply a poor prognosis. Laboratory examinations, such as leucocyte and platelet count and serum albumin level, may be potential predictors of mortality in these patients.

COVID-19-related mortality rate ranges from 1.4 to 8% in the general population. A recently published meta-analysis of 29 international studies demonstrated that the overall mortality rate was 22.4%, and fever was the predominant clinical manifestation in HD patients with COVID-19 [42]. However, their study did not further investigate the risk factors for mortality between surviving and non-surviving HD patients. Most HD patients were old and had multiple comorbidities, such as hypertension, diabetes, and cardiovascular disease. Because of the uremic status, HD patients tend to have a weaker immune system with increased susceptibility to infections [43]. In addition, the HD room where the patients had to visit three times weekly was a crowded and enclosed space, which increased the risk of disease transmission.

CKD is an independent risk factor for COVID-19-associated in-hospital mortality in elderly patients, and acute-on-chronic kidney injury increases the odds of in-hospital mortality in patients with CKD hospitalized with COVID-19 [44]. A study showed that compared with patients without preexisting CKD, dialysis patients had a higher risk for 28-d in-hospital death, whereas patients with non-dialysis-dependent CKD had an intermediate risk [45]. Our data showed that in HD patients, males tend to have higher mortality than females, which might be associated with lifestyle and underlying diseases. As immunity and organ function declines with age, elderly HD patients are more likely to die. These results are similar to those of previous studies in the general population [46]. Interestingly, we found that HD patients with glomerulonephritis as the primary ESRD have a better prognosis than those with diabetes and hypertension. In addition, a previous study reported that other patients with comorbidities could have increased risk of COVID-19-related mortality [47,48]. Our study also indicated that cardiovascular and respiratory diseases were associated with higher risk of COVID-19-related mortality in HD patients. Patients with cardiovascular or respiratory disease have weakened cardiac or pulmonary function, which makes them more likely to have acute cardiovascular events or develop ARDS; thus, they were considered risk factors for disease progression. However, hypertension and diabetes were shown to be risk factors in the general population and are probably not predictors of mortality in HD patients.

COVID-19 patients with CKD have a high incidence of neutrophilia, poor prognosis, and in-hospital death, with dialysis patients being more vulnerable [49]. The most common clinical symptoms of COVID-19 are fever, cough, dyspnea, and diarrhea, which are the same in HD and non-HD patients [50–53]. A European study identified that infection-related pulmonary symptoms, such as fever, cough, and dyspnea, were more prevalent in patients with moderate-to-severe COVID-19 [54]. Another study also revealed that fever and cough were risk factors for deterioration in COVID-19 patients [55]. In our meta-analysis, we found that fever, cough, and dyspnea were risk factors for death in HD patients with COVID-19. On one hand, patients with these infection-related respiratory symptoms have poor lung function and low oxygen levels. On the other hand, cough and dyspnea could be the main symptoms of hypervolemia, which is frequently encountered in HD patients. Similar to previous studies in the general population, we also found that higher leucocyte and platelet count, and hypoalbuminemia were associated with higher mortality rate in HD patients [56–60]. Platelet activation plays an important role in inflammation [61]. Studies have shown that a low level of platelets contributed to COVID-19 severity [62,63]. Damaged lung tissues would cause platelet activation and thrombi formation, which lead to the consumption of platelets [64]. When leucocyte count increases, they may be associated with bacterial co-infection that aggravates the disease [65,66]. In HD patients, albumin is an indicator of a patient’s nutritional status and is related to the malnutrition–inflammation complex syndrome, which is also an important risk factor for cardiovascular mortality [67,68].

Our study has several limitations. All of the included studies were retrospective in design. The included observational studies were subject to potential confounders that may weaken or strengthen the overall results. The included studies had a relatively small sample size and short follow-up time compared with the course of the disease. Data on D-dimer, C-reactive protein, procalcitonin, and interleukin 6 levels were insufficient in the included studies and could not be analyzed. Furthermore, most studies did not provide adequate information regarding the adjusted results of risk factors. Our meta-analysis did not obtain information, such as body mass index, drinking history, and smoking history, which are also potential risk factors for disease severity and mortality. Finally, moderate heterogeneity in the range of symptoms and comorbidities across different studies could be due to demographic differences, statistical methods, follow-up duration, and the risk factors analyzed. Subgroup analysis and sensitivity analysis could only explain the source of heterogeneity to a certain extent. We further used the random-effects model for the meta-analyses that were analyzed in a fixed-effect model to strengthen our study and enhance the reproducibility of the results. The conclusions of this meta-analysis still need to be verified by more relevant studies with larger sample sizes, more careful design, and more rigorous implementation. Despite these limitations, our meta-analysis has several advantages. First, to the best of our knowledge, this is the first meta-analysis to identify the clinical risk factors for fatal outcomes in HD patients with COVID-19. In addition, the heterogeneity across the studies was mostly low or moderate, which enhanced the reliability of our results.

In conclusion, male patients might have a higher risk of developing severe COVID-19. Comorbidities, such as respiratory diseases could also greatly influence the clinical prognosis of COVID-19. Clinical features, such as fever, dyspnea, cough, and abnormal platelet, leucocyte, and albumin levels could imply eventual death. Our findings will help clinicians identify markers for the detection of high mortality risk in HD patients at an early stage of COVID-19.

## Supplementary Material

Supplemental Material

Supplemental Material

Supplemental Material

## Abbreviations

HD: hemodialysis
COVID-19: coronavirus disease 2019
SARS-CoV-2: severe acute respiratory syndrome coronavirus 2
ESRD: end-stage renal disease
QUIP S: Quality In Prognosis Studies
CI: confidence intervals
WMD: weighted mean difference
