# Supplementary material for: Risk factors for mortality in hemodialysis patients with COVID-19: a systematic review and meta-analysis
Source: Ren Fail. 2021 Oct 11;43(1):1394–407. doi: 10.1080/0886022X.2021.1986408 (PMC8510603; doi:10.1080/0886022X.2021.1986408)
Supplement: Supplemental Material [file IRNF_A_1986408_SM2689.pdf]

Online Supplementary Material

Supplementary Material. Systematic Review search strategy used for PubMed, EMBASE and Cochrane databases

Pubmed

Search Strategy:

(((((((((hemodialysis) OR (renal replacement therapy)) OR (dialysis)) OR (renal insufficiency)) OR (haemodialysis)) OR (chronic kidney disease)) OR (chronic kidney failure)) OR (CKD-G5D)) OR (end-stage kidney disease)) OR (hemodialysis[MeSH Terms])) OR (renal insufficiency, chronic [MeSH Terms])) OR (end-stage renal disease[MeSH Terms])) OR (renal replacement therapy[MeSH Terms])) OR (dialysis[MeSH Terms])) OR (kidney failure, chronic[MeSH Terms])) AND (((((((((2019-nCoV) OR (coronavirus disease 2019)) OR (COVID-19)) OR (SARS-CoV-2)) OR (novel coronavirus)) OR (COVID-19[MeSH Terms])) OR (coronavirus disease 2019[MeSH Terms])) OR (2019-nCoV[MeSH Terms])) OR (novel coronavirus[MeSH Terms]))

Cochrane

Search Strategy:

ID	Search
#1	novel coronavirus
#2	MeSH descriptor: [SARS-CoV-2] explode all trees
#3	coronavirus disease 2019
#4	MeSH descriptor: [COVID-19] explode all trees

#5 SARS-CoV-2

#6 COVID-19

#7 #1 or #2 or #3 or #4 or #5 or #6

#8 hemodialysis

#9 MeSH descriptor: [Renal Dialysis] explode all trees

#10 renal insufficiency

#11 MeSH descriptor: [Renal Insufficiency] explode all trees

#12 end-stage renal disease

#13 MeSH descriptor: [Kidney Failure, Chronic] explode all trees

#14 renal replacement therapy

#15 MeSH descriptor: [Renal Replacement Therapy] explode all trees

#16 dialysis

#17 MeSH descriptor: [Dialysis] explode all trees

#18 chronic kidney disease

#19 MeSH descriptor: [Renal Insufficiency, Chronic] explode all trees

#20 chronic kidney disease

#21 CKD-G5D

#22 MeSH descriptor: [Kidney Failure, Chronic] explode all trees

#23 #8 or #9 or #10 or #11 or #12 or #13 or #14 or #15 or #16 or #17 or #18 or #19 or #20 or #21or #22

#24 #7 and #23

Embase

Search Strategy:

1 novel coronavirus.mp. [mp=title, abstract, original title, name of substance word, subject heading word, floating sub-heading word, keyword heading word, organism supplementary concept word, protocol supplementary concept word, rare disease supplementary concept word, unique identifier, synonyms]

2 2019-nCoV.mp. [mp=title, abstract, original title, name of substance word, subject heading word, floating sub-heading word, keyword heading word, organism supplementary concept word, protocol supplementary concept word, rare disease supplementary concept word, unique identifier, synonyms]

3 coronavirus disease 2019.mp. [mp=title, abstract, original title, name of substance word, subject heading word, floating sub-heading word, keyword heading word, organism supplementary concept word, protocol supplementary concept word, rare disease supplementary concept word, unique identifier, synonyms]

4 SARS-CoV-2.mp. [mp=title, abstract, original title, name of substance word, subject heading word, floating sub-heading word, keyword heading word, organism supplementary concept word, protocol supplementary concept word, rare disease supplementary concept word, unique identifier, synonyms]

5 COVID-19.mp. [mp=title, abstract, original title, name of substance word, subject heading word, floating sub-heading word, keyword heading word, organism supplementary concept word, protocol supplementary concept word, rare disease supplementary concept word, unique identifier, synonyms]

6 exp Betacoronavirus/ or exp SARS-CoV-2/ or exp Pandemics/ or exp Severe Acute Respiratory Syndrome/ or exp Humans/ or exp Pneumonia, Viral/ or exp Coronavirus Infections/ or exp COVID-19/

7 exp SARS-CoV-2/

8 exp COVID-19/

9 1 or 2 or 3 or 4 or 5 or 6 or 7 or 8

10 hemodialysis.mp. [mp=title, abstract, original title, name of substance word, subject heading word, floating sub-heading word, keyword heading word, organism supplementary concept word, protocol supplementary concept word, rare disease supplementary concept word, unique identifier, synonyms]

11 renal insufficiency.mp. [mp=title, abstract, original title, name of substance word, subject heading word, floating sub-heading word, keyword heading word, organism supplementary concept word, protocol supplementary concept word, rare disease supplementary concept word, unique identifier, synonyms]

12 end-stage renal disease.mp. [mp=title, abstract, original title, name of substance word, subject heading word, floating sub-heading word, keyword heading word, organism supplementary concept word, protocol supplementary concept word, rare disease supplementary concept word, unique identifier, synonyms]

13 renal replacement therapy.mp. [mp=title, abstract, original title, name of substance word, subject heading word, floating sub-heading word, keyword heading word, organism supplementary concept word, protocol supplementary concept word, rare disease supplementary concept word, unique identifier, synonyms]

14 dialysis.mp. [mp=title, abstract, original title, name of substance word, subject heading word, floating sub-heading word, keyword heading word, organism supplementary concept word, protocol supplementary concept word, rare disease supplementary concept word, unique identifier, synonyms]

15 haemodialysis mp. [mp=title, abstract, original title, name of substance word, subject heading word, floating sub-heading word, keyword heading word, organism supplementary concept word, protocol supplementary concept word, rare disease supplementary concept word, unique identifier, synonyms]

16 chronic kidney disease mp. [mp=title, abstract, original title, name of substance word, subject heading word, floating sub-heading word, keyword heading word, organism supplementary concept word, protocol supplementary concept word, rare disease supplementary concept word, unique identifier, synonyms]

17 chronic kidney failure mp. [mp=title, abstract, original title, name of substance word, subject heading word, floating sub-heading word, keyword heading word, organism supplementary concept word, protocol supplementary concept word, rare disease supplementary concept word, unique identifier, synonyms]

18 CKD-G5D mp. [mp=title, abstract, original title, name of substance word, subject heading word, floating sub-heading word, keyword heading word, organism supplementary concept word, protocol supplementary concept word, rare disease supplementary concept word, unique identifier, synonyms]

19 end-stage kidney disease mp. [mp=title, abstract, original title, name of substance word, subject heading word, floating sub-heading word, keyword heading word, organism supplementary concept word, protocol supplementary concept word, rare disease supplementary concept word, unique identifier, synonyms]

20 exp Renal Dialysis/

21 exp Renal Insufficiency/

22 exp Renal Replacement Therapy/

23	exp Dialysis/
24	exp Chronic kidney failure/
25	exp Haemodialysis/
26	exp End-stage kidney disease/
27	10 or 11 or 12 or 13 or 14 or 15 or 16 or 17 or 18 or 19 or 20 or 21 or 22 or 23 or 24 or 25 or 26
21	9 and 27
