# Supplementary material for: Risk factors for mortality in hemodialysis patients with COVID-19: a systematic review and meta-analysis
Source: Ren Fail. 2021 Oct 11;43(1):1394–407. doi: 10.1080/0886022X.2021.1986408 (PMC8510603; doi:10.1080/0886022X.2021.1986408)
Supplement: Supplemental Material [file IRNF_A_1986408_SM2671.pdf]

Supplemental material 2

Funnel plots and Contour-enhanced funnel plot assessing publication bias

1 Diabetes

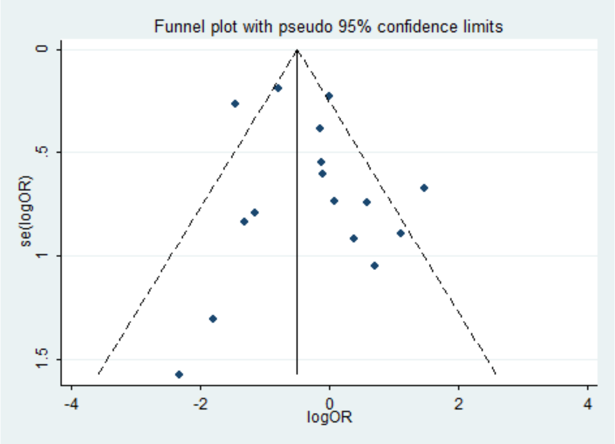

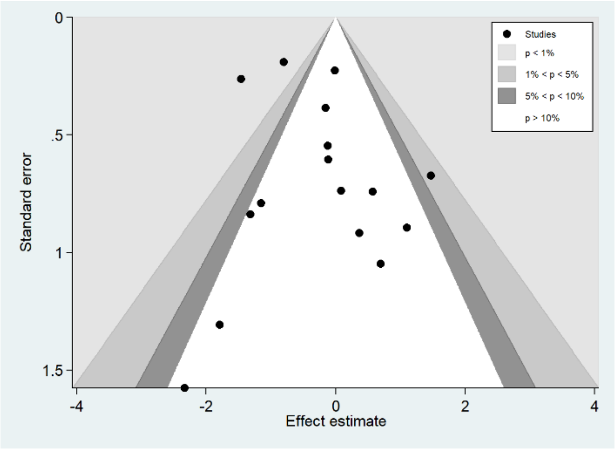

Begg's Test, p=0.822
Egger's test, p= 0.276

2 Hypertension

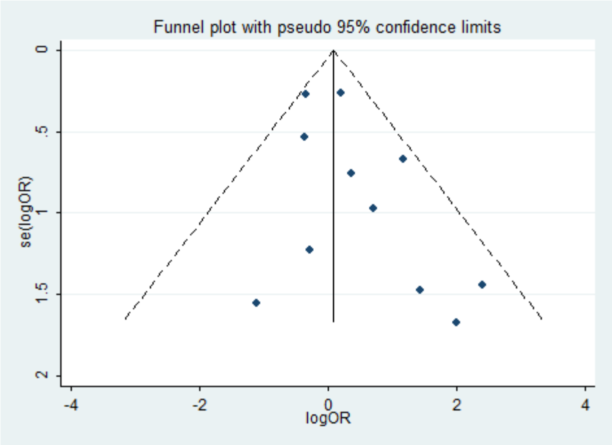

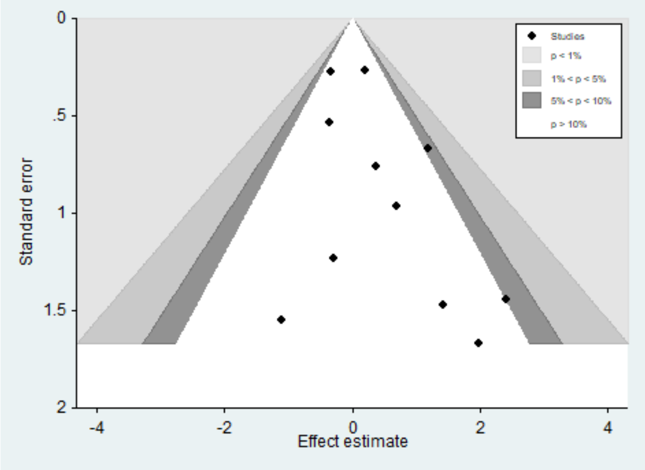

Begg's Test, p= 0.35
Egger's test, p= 0.118

3 Cardiovascular disease

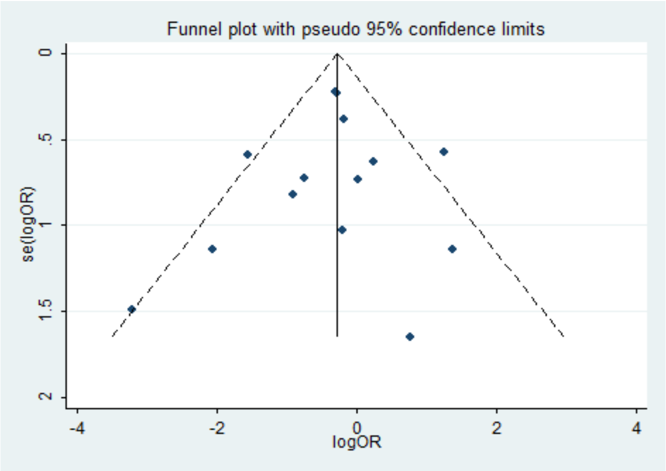

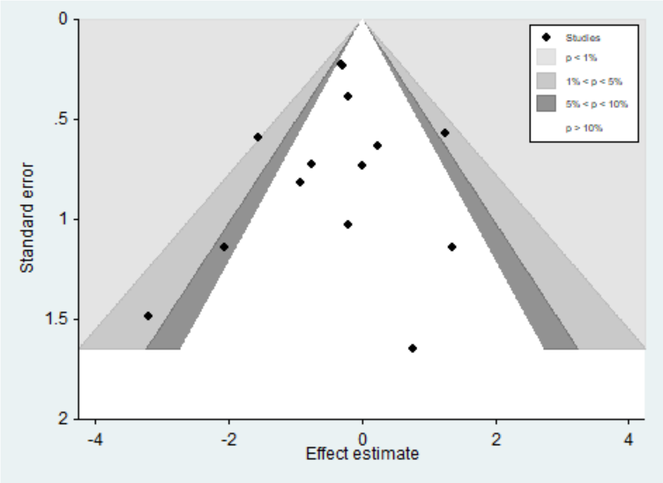

Begg's Test, p= 0.827
Egger's test, p= 0.733

4 Fever

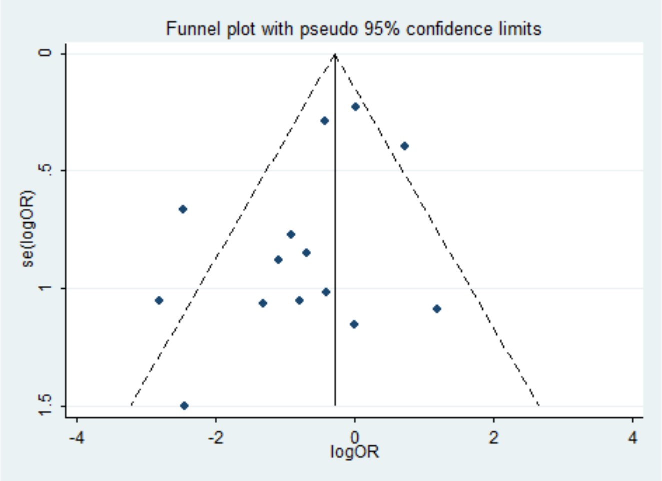

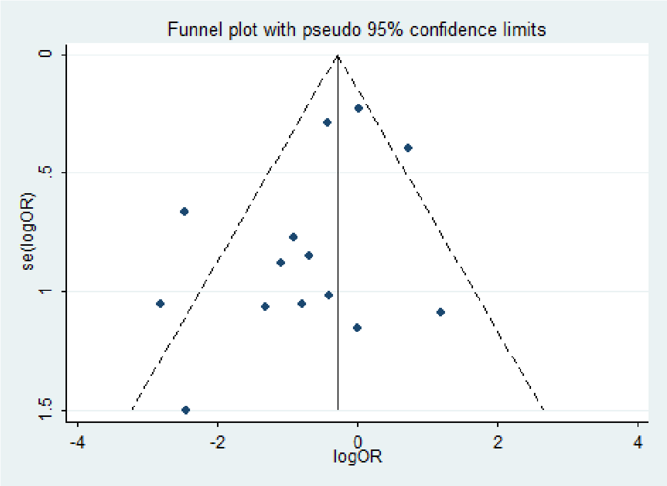

Begg's Test, p= 0.511
Egger's test, p= 0.081

5 Cough

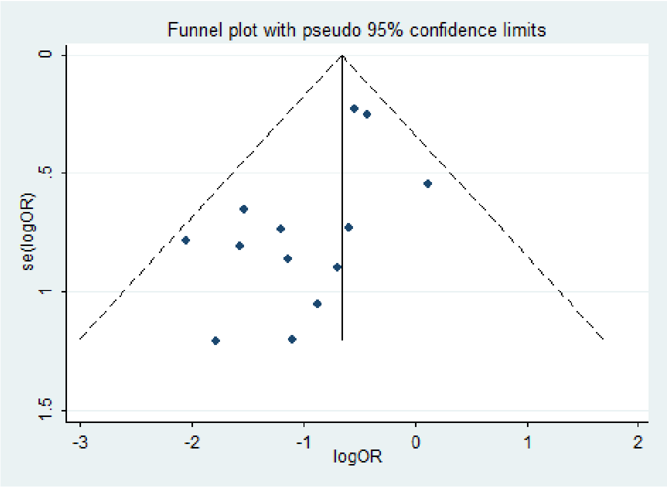

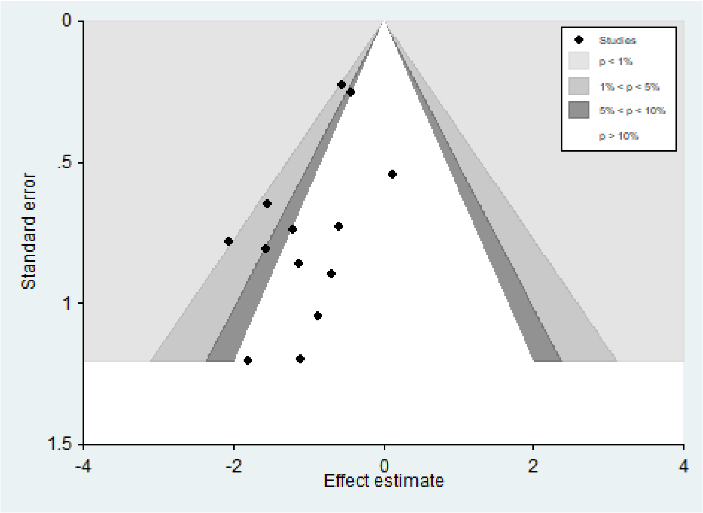

Begg's Test, p= 0.246
Egger's test, p= 0.025

6 Diarrhea

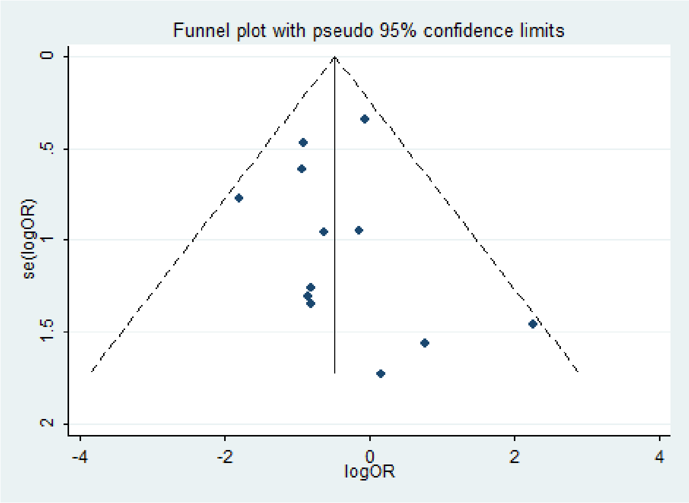

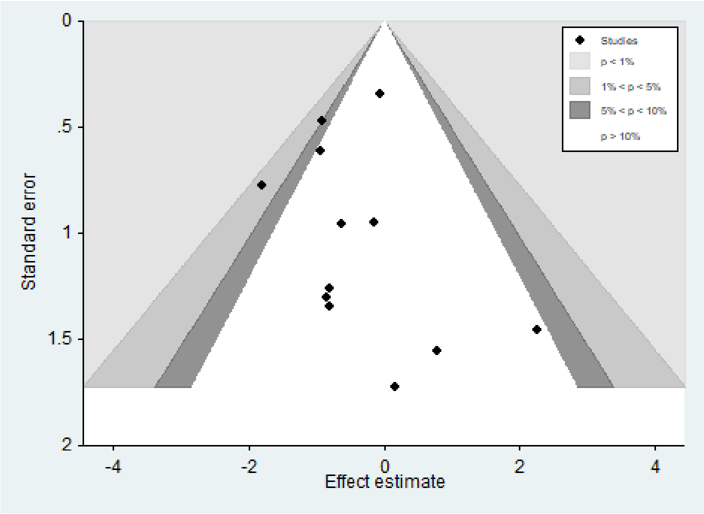

Begg's Test, p= 0.193
Egger's test, p= 0.809

7 Male

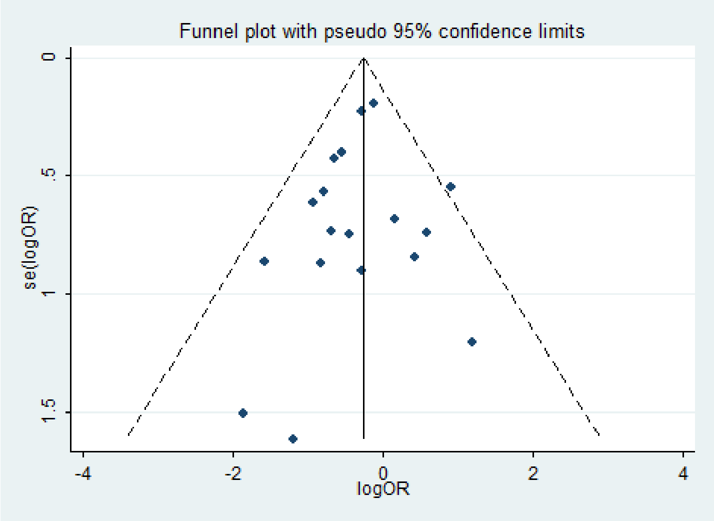

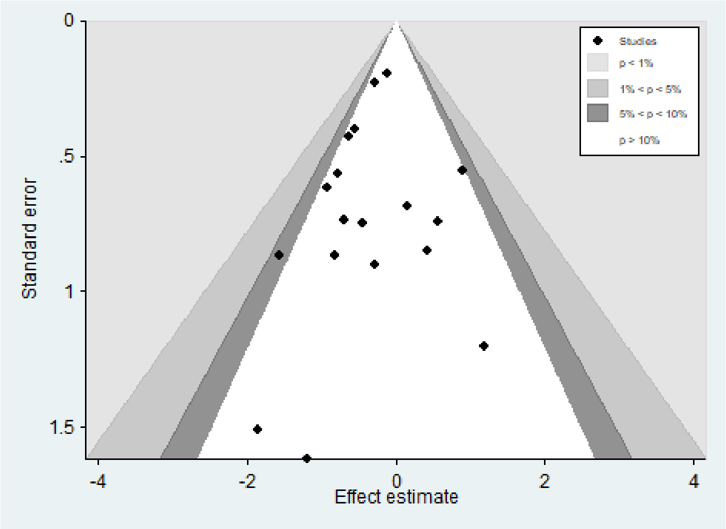

Begg's Test, p= 0.762

Egger's test, p= 0.505

8 Age

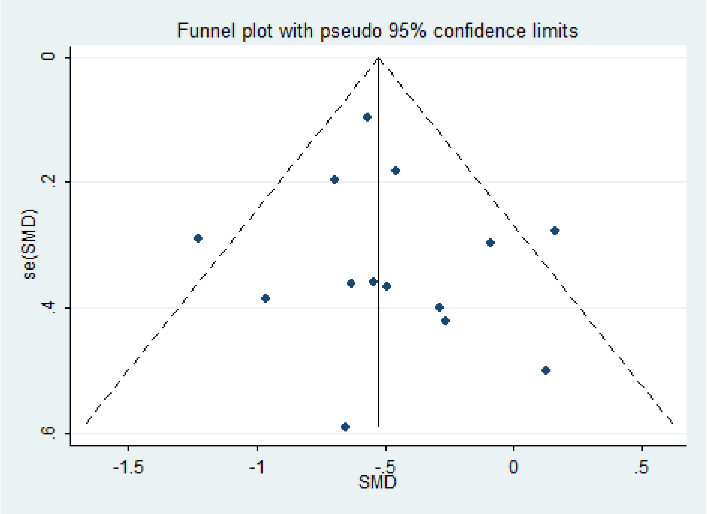

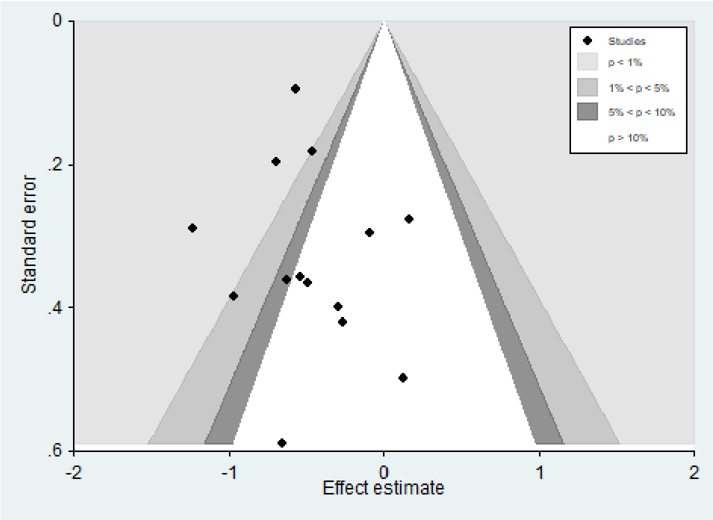

Begg's Test, p= 0.511
Egger's test, p= 0.543
